# 961. Experience, Lessons, and Strategies in Developing a High-Impact Real-Time Learning Network for Clinicians Caring for Patients with COVID-19 Infection

**DOI:** 10.1093/ofid/ofab466.1156

**Published:** 2021-12-04

**Authors:** Ravina Kullar, Payal K Patel, Marjorie Connolly, Coran Jallah, Gayle Levy, Varun Phadke, Varun Phadke, Ethel Weld, William Werbel, Andrea Weddle, Dana Wollins, Natasha Chida

**Affiliations:** 1 Expert Stewardship Inc., LA, California; 2 University of Michigan and the Ann Arbor VA Healthcare System, Ann Arbor, MI; 3 Humboldt University of Berlin, Berlin, Brandenburg, Germany; 4 IDSA, Arlington, Virginia; 5 Emory University, Atlanta, GA; 6 Johns Hopkins, Baltimore, Maryland; 7 Johns Hopkins University School of Medicine, Baltimore, Maryland

## Abstract

**Background:**

Accurate and rapid dissemination of clinical information is vital during pandemics, particularly with novel pathogens. To respond to the high volume and constantly evolving knowledge during the COVID-19 pandemic, the Infectious Diseases Society of America (IDSA) created an online educational COVID-19 Resource Center for frontline clinicians.

**Methods:**

In February 2020, IDSA launched an online resource center for COVID-19, which housed relevant clinical guidance, institutional protocols, and clinical trials. Then, in September 2020, IDSA leveraged a CDC grant to transform the resource center into the COVID-19 Real Time Learning Network (RTLN), a user-friendly, up-to-date microsite that contains clinically focused original content, guidelines, resources, and multimedia (Figure 1). The RTLN is supported by a team consisting of a Medical Editor, Associate Editors, an Online Editor, and IDSA staff. As of June 2021, the RTLN housed 12 sections, 7 of which are comprised of original content; these 7 sections contain a total of 37 subsections. A Twitter account (@RealTimeCOVID19) was also created in October 2020 to share information from RTLN in real-time.

Figure 1. COVID-19 Real Time Learning Network Microsite

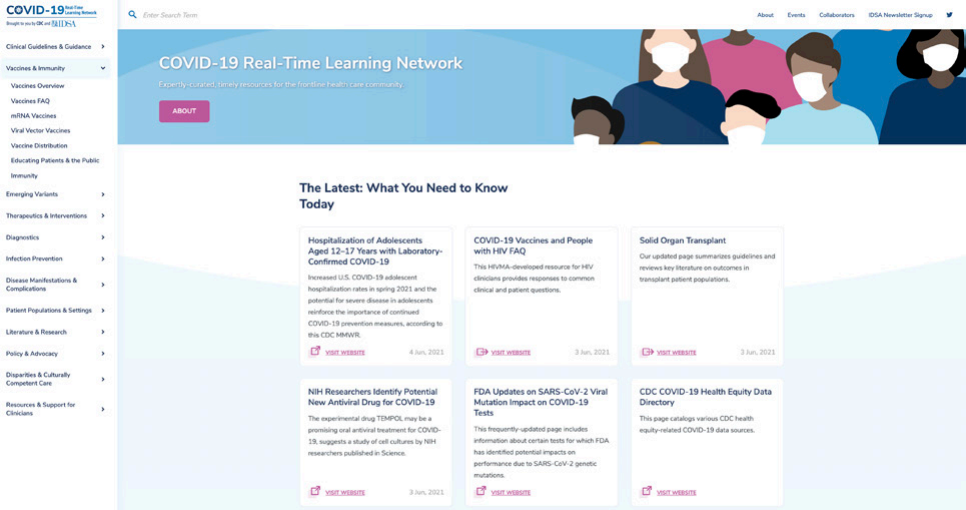

**Results:**

As of June 2021, the most visited page of the RTLN was the Moderna Vaccine page, with 486,969 page views (Figure 2). Peak monthly page views are displayed in Figure 3. Between October 2020 and June 2021, the RTLN Twitter account had 2,911 followers, 2,135,783 impressions, and 41,793 engagements. The account had also hosted 2 Twitter Chats on COVID-19 vaccines; these chats resulted in 19 million and 5.3 million impressions, respectively. Twitter engagements by month are displayed in Figure 4.

Figure 2. Literature Review of Moderna COVID 19 Vaccine on RTLN

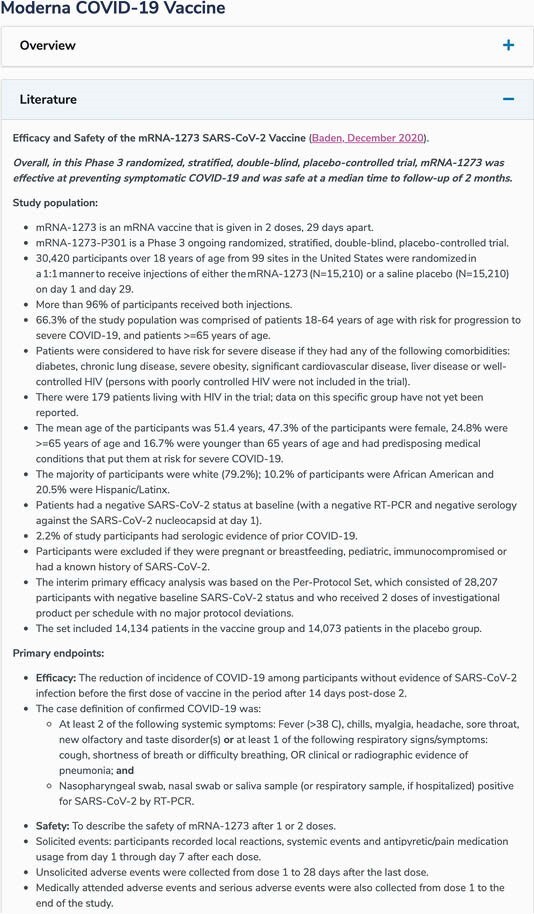

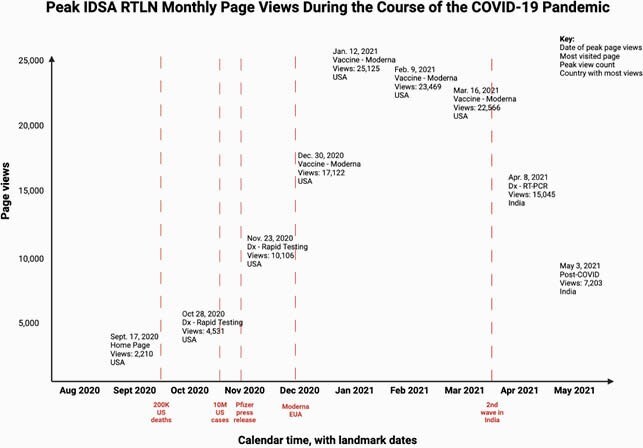

Figure 4. RTLN Twitter Engagements By Month

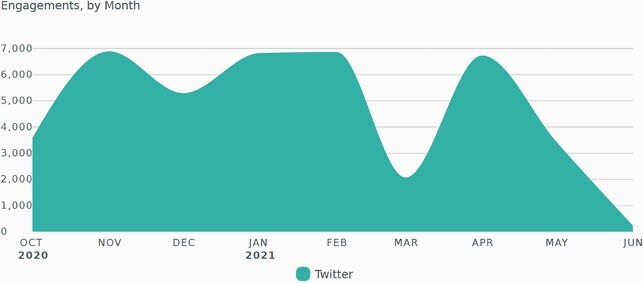

**Conclusion:**

A comprehensive educational microsite housing clinically relevant COVID-19 information had high uptake, and an accompanying Twitter account had significant engagement. Rapid curation is labor-intensive and required expansion of our editorial team. To ensure we continue to serve the needs of our users a qualitative survey is planned. Our experience launching the RTLN can serve as a roadmap for the development of accessible and nimble educational resources during future pandemics.

**Disclosures:**

**Varun Phadke, MD**, Nothing to disclose

